# A Systematic Approach to Developing Virtual Patient Vignettes for Pediatric Health Equity Research

**DOI:** 10.1089/heq.2022.0108

**Published:** 2022-11-22

**Authors:** Siddika S. Mulchan, Megan Miller, Christopher B. Theriault, William T. Zempsky, Adam Hirsh

**Affiliations:** ^1^ Center for Cancer and Blood Disorders, Connecticut Children's,Hartford, Connecticut, USA.; ^2^ Division of Pain and Palliative Medicine, Connecticut Children's, Hartford, Connecticut, USA.; ^3^ Division of Behavioral Medicine and Clinical Psychology, Cincinnati Children's Hospital Medical Center, Cincinnati, Ohio, USA.; ^4^ Department of Pediatrics, University of Cincinnati College of Medicine, Cincinnati, Ohio, USA.; ^5^ Department of Pediatrics, University of Connecticut School of Medicine, Farmington, Connecticut, USA.; ^6^ School of Science, Indiana University–Purdue University Indianapolis, Indianapolis, Indiana, USA.

**Keywords:** health equity, pediatrics, pain management, virtual human technology

## Abstract

**Objective:**

The aim of this study was to describe a systematic approach to developing virtual patient (VP) vignettes for health equity research in pediatric pain care.

**Methods:**

VPs were initially developed to depict the body posture and movements of actual children experiencing pain. Researchers and clinicians with expertise in pediatric pain worked closely with a professional animator to portray empirically supported pain expression in four, full-motion, virtual male characters of two races (i.e., White and Black). Through an iterative process, VPs were refined to (1) appear realistic in a clinical setting and (2) display archetypal pain behavior and expression during a 1-min video clip without sound. Text vignettes were developed with consultation from experts in pain care and presented alongside VPs to assess clinical decision-making. VP vignettes were piloted in a sample of pediatric providers (*N*=13).

**Results:**

Informed by the literature and expertise of stakeholders, several revisions were made to improve VPs' facial grimacing and realism before piloting. VPs appeared to accurately capture important aspects of pain expression and behavior common among pediatric patients with pain disorders. Additional refinements to the text vignettes were made based on provider feedback to improve clarity and clinical relevance.

**Conclusions:**

This article presents a working framework to facilitate a systematic approach to developing VP vignettes. This framework is a first step toward advancing health equity research by isolating psychosocial and interpersonal factors affecting provider behavior and decision-making. Future research is needed to validate the use of VP vignettes for assessing provider behavior contributing to health inequities for youth with pain disorders.

## Introduction

Health equity has become a priority among several national health care organizations^1,2^ as research continues to expose inequities for marginalized populations.^3^ This field has recently extended to pediatric health care as disparities, particularly in pain care, have been documented for children and adolescents of color compared with White youth.^4,5^ One mechanism through which treatment disparities are perpetuated is when health care provider behavior and decision-making systematically differ across patient demographic characteristics.^6,7^

However, a nuanced understanding of this mechanism has yet to be ascertained. Contributing to challenges elucidating the relationship between provider behavior, specifically differential decision-making, and health inequities is the lack of validated measures designed to assess provider bias.^8^

The case vignette methodology has been used broadly in health care research to facilitate training, education, and clinical decision-making. This methodology typically involves presenting participants with a written stimulus that can be manipulated by researchers to evaluate factors affecting internal processes, such as thoughts, feelings, behaviors, and decision-making.^9^

Research has documented that case vignettes can be applicable to real-world situations.^10,11^ The representativeness of written case vignettes has been further amplified by technological advances in the area of virtual patients (VPs).^12^ VPs can be designed and presented as medical patients to accompany text vignettes, comprising VP vignettes.

Compared with patient simulation by human actors and text vignettes alone, VPs allow greater experimental control and ecological validity, and participants' responses to them are less likely to be influenced by social desirability bias.^13^ VPs have been used specifically to examine bias and health-related stigma in previous research, particularly in the domain of pain assessment.

For example, several studies manipulated patient demographics and other contextual cues (e.g., high or low pain expression) using VPs to evaluate pain treatment disparities.^4,12,14–16^ These studies utilized a web-based platform (e.g., Qualtrics) to present VPs to participants who then completed visual analog scales (VASs) and other self-report measures assessing their beliefs and pain treatment decisions. VP profiles typically consisted of a static image or brief video accompanied by a text vignette.

VP videos were developed with various software systems (e.g., *People Putty*, Lifelike Responsive Avatar Framework) and systematically manipulated using the Facial Action Coding System (FACS)^17^ as a guide. Text vignettes included clinical information and physiological data (e.g., patient temperature, blood pressure, pulse rate, respiration rate, and mental status), which were typically held constant across the VP cues of interest (e.g., race, gender, age).

In the majority of these studies, researchers sought to optimize external validity by presenting VP vignettes in a format consistent with the clinical environment. For example, one study interested in the pediatric intensive care unit (PICU) setting presented physiological data in a visual format (e.g., video monitor of vital signs, screenshots of electronic medical records).^16^

Another study on pediatric pain included VP child–parent dyads in the emergency department (ED).^4^ Some studies employed a predetermined profile-to-cue ratio resulting in VPs being presented at least twice to enhance statistical power.^12,14,15^

While the use of VPs appears to be burgeoning in health care research, few studies detail the methodology used to develop these stimuli and accompanying text vignettes. Indeed, the process of developing text vignettes often lacks a systematic approach,^18^ as evidenced by the fact that these are commonly written by a member of the research team who may have clinical experience, but not the methodological expertise necessary to ensure scientific rigor.^19^

Of the studies reviewed, only two articles provided a list of criteria for developing text vignettes.^9,20^ The development process for VPs also varies considerably across studies, with few providing a detailed description of how the characters were developed for research purposes.^13,14,16,21^

The objective of this methodological article is to describe a systematic approach to developing VP vignettes for health equity research in pediatric pain care. We first describe the development process for creating videos and text vignettes depicting four adolescent male VPs presenting to the ED with pain associated with sickle cell disease (SCD) or cancer. Then, to illustrate the use of this methodological approach, we present quantitative data and written feedback from a pilot study with pediatric providers.

Finally, we provide a working framework to guide the future development of VPs for use in health equity research focused on provider behavior and decision-making in pediatric pain care.

## Materials and Methods

This study was conducted at a mid-sized, freestanding children's hospital in the Northeast. Approval from the hospital's Institutional Review Board was obtained. VPs were initially developed through an iterative process (described below) before the accompanying text vignettes. The text vignettes were written according to recommendations published in the literature.^9^

For the purpose of clarification, the term “VP vignettes” will be used throughout this article to indicate the VP video together with the accompanying text vignette. Next, VP vignettes were piloted among a sample of pediatric hospitalists and medical residents (*N*=13) and refined based on their feedback. This systematic pilot was conducted as part of a larger study examining health care providers' racial bias on clinical decision-making in pediatric SCD pain compared with cancer pain.

We first describe the development of VP vignettes below, then present results of our pilot study.

### Development of VP vignettes

We convened an initial meeting with an expert in human-centered computing who confirmed that virtual human technology was appropriate for study variables (i.e., provider bias, pediatric pain care) and facilitated communication with a professional three-dimensional animator.

#### Virtual patients

##### Character design

VPs were developed from existing models of Black and White adolescent males used in previous research examining provider decision-making in pediatric patients with chronic abdominal pain.^4^ Three Black virtual characters and one White virtual character were selected for modification based on the following predetermined parameters: male gender, appearance congruent with a 16-year-old, in the hospital room of an ED setting, and unaccompanied by parent/caregiver.

Each parameter was set with a specific purpose. Male gender was selected due to previous research indicating that health care providers may perceive males as experiencing higher pain than females.^22^ Sixteen years of age and the ED setting were selected given that SCD-related pain often worsens during adolescence and young adulthood, resulting in increased emergent encounters for pain crises.^23^

The 3:1 Black-to-White character ratio was selected to develop a robust stimulus set of Black characters given the study's focus on racialized groups. Additionally, a Black character with acute lymphoblastic leukemia (ALL) was included primarily to control for diagnosis vs. race as a factor affecting provider decision-making.

No parent/caregiver was included with VPs to reduce potential confounding affecting providers' pain assessment and decision-making. Additional characteristics that were held constant included body posture, clothing, length of video, and timing of facial grimacing. Two of the virtual characters (one Black and one White) were designed to have close-cropped hair due to their intended depiction as patients with ALL for the parent study comparing SCD with cancer pain treatment.

##### Pain animation

Publicly available online images and videos of adolescents with SCD, experiencing pain crises, obtained through web-based search engines (e.g., Google) and video sharing websites (e.g., YouTube) were shared with the animator to facilitate VP design and presentation. The initial VP (i.e., Black male intended to have SCD) was modified through an iterative process and based on verbal feedback provided by multiple stakeholders (two young adult patients with SCD, four parents/caregivers of patients with SCD, one hematology nurse, and one hematologist) and the study team.

**FIG. 1.**
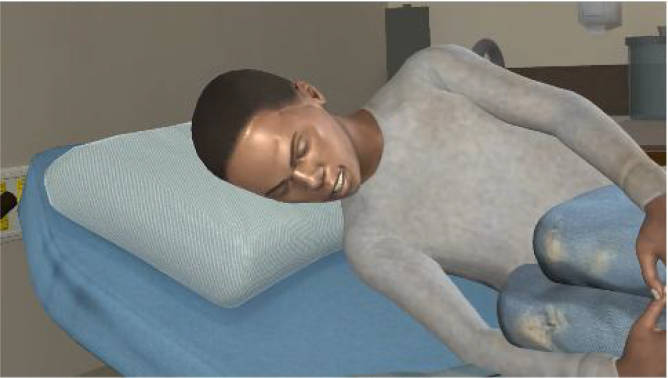
Still-frame of pain expression for a Black virtual patient.

**FIG. 2.**
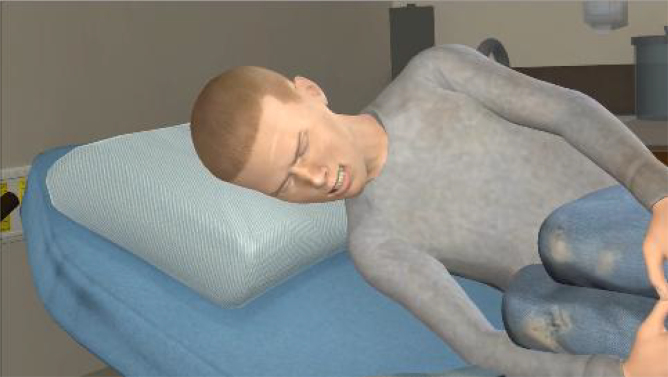
Still-frame of pain expression for a White virtual patient.

The pain animation begins with a full view of VPs lying on their side in the fetal position on a bed in an ED hospital room. After 10 sec, the animation zooms in on the patient's facial expression to provide a more realistic depiction of the character's pain expression from the perspective of a clinician during a patient encounter. See Figures 1 and 2 for still-frame images of the pain expression for Black and White VPs, respectively.

##### Final VPs

Based on stakeholder feedback and communication between the study team and the animator, modifications to VPs included increasing the hair length and softening facial lines to create a more youthful appearance, increasing facial grimacing, and adding perspiration, lip quivering, and a tissue box on the hospital bed. Once the body posture, facial characteristics, pain expression, and background environment of the first character were deemed satisfactory and consistent with stakeholder feedback and study team deliberations, this model was used as a template to create the subsequent three characters.

The second character (i.e., White male intended to have ALL) required adjustments such as closely cropped hair, removal of facial lines contributing to a smiling countenance, and changes to the mouth to refine the intensity of pain expression. The third and fourth characters required minimal changes.

#### Text (written) vignettes

Accompanying text vignettes were presented immediately below looped videos of the VPs on the same screen. Text vignettes, treatment recommendations, and response options (see Treatment Recommendations and Response Options) were developed in consultation with clinical and research experts in SCD pain care. They were intended to provide information routinely obtained by providers through medical record review and a physical examination.

Physiological data included height, weight, presenting problem (pain), temperature, respiration rate, heart rate, blood pressure, and mental status (alert and oriented×4). These data varied slightly, but in terms of clinical significance, they were consistent across vignettes.

Text summarizing the history and physical examination was developed using guidelines from Evans et al^9^ and previous case vignette research assessing provider bias in pediatric care.^4,19,24^ The text included a subjective pain rating on a scale from 1 to 10 (10 being the highest pain), which was also held constant at 8 of 10 for each VP vignette.

VP names were selected based on publicly available census data on the most common first and last names among Black and White Americans.^25,26^ Age varied between 16 and 17 years.

##### Treatment recommendations and response options

After each text vignette, two possible treatment recommendations were presented with two responses for each recommendation (total of four response options). Treatment recommendations focused on various aspects of pain care and included (1) the route of administration of pain medication (i.e., oral vs. intravenous, time-controlled vs. patient-controlled analgesia) and (2) discharge planning (i.e., with vs. without prescription pain medication).

Recommendations and response options varied to align with the scope of practice for participating providers (i.e., physician/advanced practice provider [APP] or nurse). In all cases, one response option was more consistent with best practices in pediatric SCD pain care. For example, the American Society of Hematology (ASH) guidelines for SCD pain management indicate that patient preferences should drive clinical decision-making.^27^ Some response options were directly counter to the text describing the VPs' requests.

See Figures 3 and 4 for a full presentation of the VP vignettes (screenshot of video, text vignette, recommendations, and response options) for physicians/APP and nurses.

**Figure:**
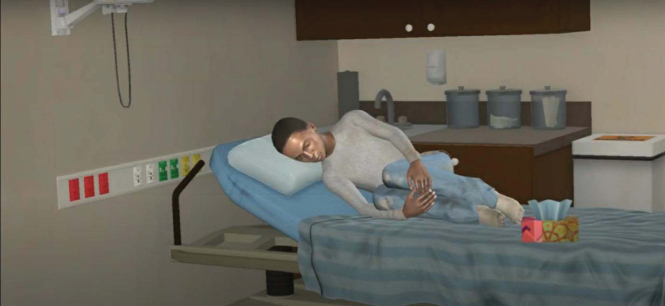
**Table:** 

Name	Age	Grade level	Height/weight	Presenting problem
John Williams	16 years	10th	5 feet 6 inches, 108 lbs	Pain

Temperature	Respiratory rate	Heart rate	Blood pressure	Mental status
98.6 F	15 rpm	66 bpm	120/80	A/O×4 John is a 16-year-old African American male with HgSS who presents to the emergency department with back pain. His pain started last night. John describes his pain as sharp and throbbing and rates it as 8 of 10. He has missed several days of school this month because of pain. He also reports sleeping difficulties and feeling tired during the day. His physical examination is unremarkable except for moderate tenderness in the lower back. His laboratory values are normal. John reports that he is completely out of his prescription pain medication (oxycodone) and says he has been taking over-the-counter medications, but none have been helpful for his pain. **Table:** 

Physicians/Advanced Practice Providers		Nurses	
Recommendation A	Recommendation B	Recommendation A	Recommendation B
Start oral oxycodone according to patient's pain plan since he has not been taking this at home and reassess in 30 min.	Start IV dilaudid according to patient's pain plan and reassess in 30 min.	Provide support and wait until further assessment by the attending provider.	Start oral oxycodone according to patient's pain plan since he has not been taking this at home and reassess in 30 min.
After an hour, John reports improvement in his pain and is receptive to discharge home.		After an hour, John reports improvement in his pain and is receptive to discharge home. He asks for prescription pain medication to take at home, but you do not see an order in his chart.	

Recommendation A	Recommendation B	Recommendation A	Recommendation B
Discharge with a prescription for 3 days of oxycodone and coordinate an outpatient appointment with the hematology clinic.	Encourage the patient to continue taking over-the-counter medications and coordinate an outpatient appointment with the hematology clinic to refill the oxycodone prescription.	Explain that his hematology team will have to refill the prescription at his next clinic appointment.	Explain that his hematology team will have to refill the prescription at his next clinic appointment, but you will check with the attending provider whether a partial prescription can be provided. **FIG. 3.** Virtual patient vignette, Black adolescent with sickle cell pain.

**Figure:**
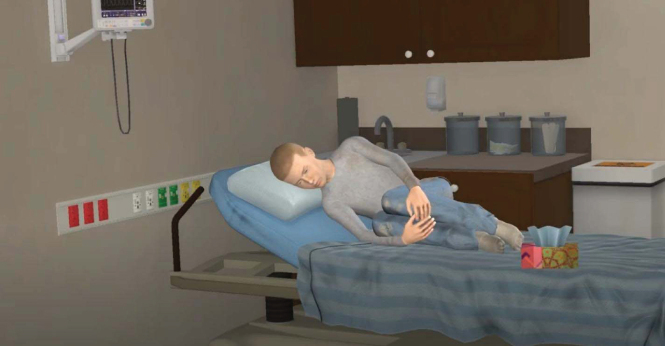
**Table:** 

Name	Age	Grade level	Height/weight	Presenting problem
Lucas Smith	17 years	11th	5 feet 6 inches, 108 lbs	Pain

Temperature	Respiratory rate	Heart rate	Blood pressure	Mental status
98.6 F	15 rpm	66 bpm	120/80	A/O×4 Lucas is a 17-year-old White male with acute lymphoblastic leukemia (ALL), on chemotherapy, who presents to the emergency department with back pain. His most recent pain reportedly started last night. Lucas describes his pain as sharp and throbbing and rates it as 8 of 10. He also reports sleeping difficulties and feeling tired during the day. His physical examination is unremarkable except for moderate tenderness in the lower back. His laboratory values are normal. Lucas reports taking prescription pain medication (dilaudid), which recently ran out, and indicates that it was not helpful for his pain. **Table:** 

Physicians/Advanced Practice Providers		Nurses	
Recommendation A	Recommendation B	Recommendation A	Recommendation B
Start oral dilaudid to address pain.	Start IV dilaudid to address pain.	Give heating packs for pain management until further assessment by the attending provider.	Provide support and wait until further assessment by the attending provider.
After an hour, Lucas reports improvement in his pain and is receptive to discharge home.		After an hour, Lucas reports improvement in his pain and is receptive to discharge home. He asks for prescription pain medication to take at home, but you do not see an order in his chart.	

Recommendation A	Recommendation B	Recommendation A	Recommendation B
Discharge with a prescription for 3 days of dilaudid and coordinate an outpatient appointment with the hematology clinic.	Coordinate an outpatient appointment with the hematology clinic to refill the dilaudid prescription.	Explain that his oncology team will have to refill the prescription at his next clinic appointment, but you will check with the attending provider whether a partial prescription can be provided.	Explain that his oncology team will have to refill the prescription at his next clinic appointment. **FIG. 4.** Virtual patient vignette, White adolescent with cancer pain.

### VP vignette pilot study

#### Procedure

Participants were recruited from the Pediatric Hospital Medicine (PHM) Department. Inclusion criteria were (1) primary work area is the PHM Department, (2) provides at least 10 h per week of direct patient care, (3) licensed or certified as a health professional or a medical resident/fellow, (4) fluent in English, (5) older than 18 years of age, and (6) able and willing to provide informed consent.

Recruitment occurred by contacting the department lead and administrative staff who were asked to share study information with eligible participants through e-mail, word of mouth, and team huddles. All prospective participants were then e-mailed a link to study measures (see Measures), which were administered through Qualtrics. Participants provided informed consent electronically by selecting “yes” before beginning the survey.

Measures could be completed at participants' convenience by a given deadline. Participants were compensated with a $20 VISA gift card.

#### Measures

##### VP vignettes

Four VP vignettes (two Black male adolescents with SCD and one Black male adolescent and one White male adolescent with cancer described above) were piloted to assess provider bias in pediatric SCD pain care. Participants were presented with videos of VPs exhibiting pain behavior in an ED hospital room, accompanied by physiological data and a text vignette.

Four relevant treatment options were presented for each vignette, of which two options were more consistent with best practices in pediatric SCD pain care. Participants rated their level of agreement with each treatment option on a 5-point Likert scale based on previous research.^24^ To reduce order bias, VP vignettes were presented randomly to each participant.

##### VAS of pain perception

The VAS in the present study was a measure designed to assess participants' perceptions of the amount of pain, emotional distress, and reaction to pain (i.e., stoicism vs. exaggeration) VPs appeared to be experiencing. Participants provided separate ratings of these factors for each VP on a 10-point VAS ranging from 0 (lowest score) to 10 (highest score).

##### VP quality ratings

Quantitative data were obtained from participants on the quality and real-life representativeness of the VPs. Participants rated each VP's facial expression, body posture, and clinical setting on a 10-point scale ranging from 0 (lowest score) to 10 (highest score). A text box was available for participants to provide optional, additional written feedback.

##### Demographic questionnaire

The demographic questionnaire was an optional self-report measure developed for the purpose of this study to describe the sample. Participants were invited to provide their age, race, ethnicity, gender identity, professional role/discipline, and years of experience in their current profession.

## Results

### Participants

Participants (*N*=13) consisted primarily of non-Hispanic White (*n*=9; 69%) cisgender females (*n*=9; 69%) with a mean age of 32 years. Nearly all (*n*=11, 85%) identified as physicians (*n*=5, 38%) or physicians in training (medical intern: *n*=4, 31%, or medical resident: *n*=2, 15%). Most (*n*=8, 62%) had 5 years or less experience in their current profession. Only one participant chose not to complete the demographic questionnaire.

### VP vignettes and VAS ratings

All participants (*N*=13) completed each of the four VP vignettes and provided VAS and quality ratings. With the exception of one recommendation for an SCD VP vignette, participants endorsed higher ratings for treatment options that were consistent with best practices for all VP vignettes.

VAS ratings of pain intensity for VPs with SCD (*MSCD_VASP_*=6.89) were lower than those for VPs with ALL (*MALL_VASP_*=7.35). VAS ratings of distress were comparable between diagnoses (*MSCD_VASD_*=7.04 and *MALL_VASD_*=7.27), and VAS ratings of reaction to pain were lower (i.e., more stoic) for VPs with SCD (*MSCD_VASR_*=5.62) in comparison with VPs with ALL (*MALL_VASR_*=6.04). See Table 1 for mean VAS ratings for each VP.

**Table 1.** Mean Visual Analog Scale Ratings for Virtual Patients with Sickle Cell Disease and Acute Lymphoblastic Leukemia

VPs	VAS ratings		
	Pain	Distress	Reaction to pain
Black SCD 1	6.69	6.62	5.23
Black SCD 2	7.08	7.46	6.00
Mean SCD	6.89	7.04	5.62
Black ALL	7.85	7.46	6.00
White ALL	6.85	7.08	6.08
Mean ALL	7.35	7.27	6.04

VAS ratings for pain and distress range from 0 (lowest pain/distress) to 10 (highest pain/distress). Ratings for reaction to pain range from 0 (stoic) to 10 (exaggerating).

ALL, acute lymphoblastic leukemia; SCD, sickle cell disease; VAS, visual analog scale; VP, virtual patient.

### Quality ratings

Participants' ratings of the quality of VPs' facial expression, body posture, and the clinical setting were all considered moderate (i.e., ratings between 5 and 7) and did not differ based on diagnosis. See Table 2 for mean quality ratings overall and by diagnosis.

**Table 2.** Mean Quality Ratings for Virtual Patients

Component	Diagnosis		Overall
	Cancer	SCD	
Facial expression	6.62	6.73	6.67
Body posture	6.85	6.77	6.81
Clinical setting	7.58	7.70	7.64

Quality ratings range from 0 (lowest quality) to 10 (highest quality).

#### Written feedback (optional)

Approximately one-third of participants (*n*=4; 31%) opted to provide written feedback. Feedback included participants' reflections on the difficulties teasing apart less pain from stoicism and recommendations to clarify the wording of the response options, elaborate on the text vignette, and include sound/audio to facilitate pain assessment.

## Discussion and Proposed Framework

The case vignette methodology is common in health care research and may be beneficial in understanding psychosocial and interpersonal factors affecting health equity for marginalized populations.^4,16,21^ In particular, this methodological approach can facilitate the identification of intervention targets, such as provider bias, to reduce disparities in clinical care.

However, to realize the promise of case vignettes incorporating virtual human technology (e.g., greater experimental control and ecological validity, reduced social desirability bias from participant responses), it is necessary to develop best practice recommendations to guide the systematic development of VP vignettes.

As an initial step, we propose a working framework, based on our findings and previous literature,^9,14,15,20,28–31^ for systematically developing and using VP vignettes in pediatric health equity research focused on provider decision-making in pain care (Table 3). The working framework provides step-by-step recommendations to guide each aspect of the development process for VP vignettes, including how to isolate study variables, develop VPs and text vignettes, and conduct pilot testing.

**Table 3.** Framework for Developing Virtual Patient Vignettes in Pediatric Health Equity Research Focused on Provider Decision-Making in Pain Care

(1) Study variables	(a) Identify study variables of interest and how each will be manipulated (e.g., gender manipulated by clothing and hair length).
	(b) Develop the profile-to-cue ratio to determine the number of vignettes needed based on study variables.^14,15^
(2) Development of VPs	(a) Incorporate patient stakeholders in developing VPs.
	(b) Identify patient demographic variables, facial expression, and body posture to depict pain commonly experienced in the target population.
	(c) Work with an animator to create the pain animation prototype.
	(d) Seek feedback on pain animation from multiple stakeholders (patients, caregivers, and providers).
	(e) Revise VPs with the animator through an iterative process.
(3) Development of text vignette	(a) Develop the written vignette in accordance with recommendations from previous research^9,20^ and with qualitative input (e.g., focus groups or interviews) from the target population.^28,29^
	(b) Standardize all relevant variables of interest (e.g., race, sex, ethnicity, name, clothing, setting, presence or absence of caregiver) and explain the rationale for each selection.
	(c) Include relevant medical and psychosocial data to maintain consistency with a clinical patient encounter, as indicated by the study setting.
(4) Pilot testing of VP vignettes	(a) Pilot test VP vignettes in either the target or an adjacent population (e.g., students if the target is health professionals) to obtain quantitative and qualitative data.
	(b) Establish content validity using cognitive interviewing techniques^30,31^ and test–retest reliability.
	(c) Refine VP vignettes based on feedback obtained before using in the initial study.

This working framework is intended to inform future studies to (1) increase standardization for developing VP vignettes, (2) enhance experimental control of key patient variables associated with disparities in provider decision-making, and (3) advance the field of pediatric health equity research by examining psychosocial and interpersonal factors affecting provider behavior and contributing to treatment disparities.

This working framework may be applicable to future research aimed at understanding factors that activate provider implicit bias and/or lead to racial disparities in pain treatment. Implicit processes are, by nature, outside of one's conscious awareness,^5^ making them difficult to assess, particularly during a clinical encounter.^8^ Moreover, using actual or simulated patients to elucidate patient demographic characteristics that activate provider implicit bias may present ethical concerns and difficulties with standardization and methodological rigor.^13^

Using VP vignettes that closely align with real-life clinical settings would advance this area of study by allowing for experimental control of patient characteristics previously found to be associated with treatment disparities (e.g., race/ethnicity, gender).

Similarly, research focused on understanding the patient perspective and how implicit bias can translate into provider behavior would benefit from this working framework. Through systematic manipulation of patient variables using VPs, provider behavior and decision-making may be assessed.

For example, patient populations who reported experiencing perceived discrimination from providers, such as patients with SCD, may have their experiences more clearly delineated by identifying provider behaviors (e.g., verbal/nonverbal communication, body language, tone of voice) that convey bias/discrimination. VPs can also be tools in the implementation of interventions to address provider racial bias and improve health equity for marginalized patient populations.^21,32^

To illustrate how this VP development process can lead to an actual study, we presented data from a pilot study comparing providers' pain care decisions for VPs with SCD and ALL. We caution against overinterpreting results of this study as there were several limitations that should be noted.

First, our study was conducted at one mid-sized, tertiary children's hospital, therefore findings may not be generalizable to other medical settings (e.g., primary care) or health care providers (e.g., adult providers).

Second, we utilized a self-selected convenience sample of pediatric hospitalists and medical trainees. The homogeneity of this group could have contributed to their VAS ratings being similar across VPs.

Finally, because this was a pilot study with limited resources, we did not create the optimal number of vignettes for high-powered hypothesis testing of key variables of interest. This also prevented us from conducting a thorough evaluation of the psychometric properties (e.g., test–rest reliability, construct validity) of our VP vignettes. However, it is important to note that the primary aims of this study were methodological and descriptive (i.e., to describe the VP development process from initial development to utilization).

Future directions of our research include revising VP vignettes based on data obtained from this pilot study in preparation for use in a larger study focused on provider racial bias in pediatric SCD pain care. Revisions include (1) modifying text vignettes and response options to improve clarity, (2) developing a profile-to-cue ratio of at least 5:1^33^ based on six cues of interest (i.e., race, diagnosis, gender, age, clinical ambiguity, and presence of a caregiver) and creating additional VPs to enhance study power, (3) exploring test–retest reliability, and (4) determining convergent validity using validated measures of racial bias (e.g., Race Implicit Association Test).

In addition to these quantitative analyses, we will initiate a rigorous qualitative investigation using semistructured individual interviews to understand providers' interpretations of and responses to VP vignettes. The content of interview questions will include strategies to improve the quality and representativeness of VPs, comprehensiveness of response options, and providers' approach to treatment decision-making. Once VP vignettes have been further refined and validated, we will use them to evaluate the efficacy of an intervention to address provider implicit racial bias in pediatric SCD pain care.

In conclusion, VP vignettes have several methodological strengths that can advance pediatric health equity research focused on pain treatment disparities. The proposed working framework is a first step toward developing best practice guidelines for the development of VPs for this type of research and presents several opportunities for future studies addressing provider behavior contributing to health inequities for youth with pain disorders.

## Abbreviations Used

ALL: acute lymphoblastic leukemia
APP: advanced practice provider
ED: emergency department
PHM: Pediatric Hospital Medicine
SCD: sickle cell disease
VAS: visual analog scale
VP: virtual patient
